# Long term positional stability of the Argus II retinal prosthesis epiretinal implant

**DOI:** 10.1186/s12886-022-02736-w

**Published:** 2023-02-16

**Authors:** Nimra Ghani, Jahnvi Bansal, Abhishek Naidu, Khurram M. Chaudhary

**Affiliations:** Department of Ophthalmology, Stony Brook University Hospital, Stony Brook, NY 11790 USA

**Keywords:** Argus II, Argus II retinal prosthesis, Retinitis pigmentosa, Implant movement, Low vision

## Abstract

**Background:**

The Argus II Retinal Prosthesis System (Second Sight Medical Products, Sylmar, California) is an epiretinal prosthesis that serves to provide useful vision to people who are affected by retinal degenerative diseases such as retinitis pigmentosa (RP). The purpose of this study was to analyze postoperative movement of the electrode array.

**Methods:**

Five patients diagnosed with profound retinal dystrophy who have undergone implantation of retinal prosthesis at Stony Brook University Hospital. Fundoscopy was performed at postoperative month 1 (M1), month 3 (M3), month 6 (M6), month 12 (M12), and month 24 (M24) visits. Fundoscopy was extracted and analyzed via NIH ImageJ. Data analysis was completed using IBM SPSS. Various lengths and angles were measured each postoperative month using ImageJ.

**Results:**

There was no significant change in distance between the optic disc and the surgical handle (length AB) over the two-year span (F = 0.196, *p* = 0.705). There was a significant change in distance of length AB over time between patients between M3 and M6 (*p* = 0.025). A repeated measures ANOVA revealed that there was statistically significant change of the optic disc-tack-surgical handle angle (**𝛾**) (M1 to M24) (F = 3.527, *p* = 0.030). There was no significant change in angle 𝜟 (the angle to the horizontal of the image), angle 𝜶 (tack-optic disc-surgical handle), and angle 𝜷 (optic-disc-surgical handle-tack).

**Conclusion:**

Our results demonstrate that there may be postoperative movement of the retinal prosthesis over time, as a statistically significant downward rotation is reported over the 2 years span. It is important, moving forward, to further study this movement and to take into consideration such movement when designing retinal implants. It is important to note that this study is limited by the small sample size, and therefore, the conclusions drawn are limited.

## Introduction

The Argus II Retinal Prosthesis System (Second Sight Medical Products, Sylmar, California) is a US Food and Drug Administration (FDA) approved epiretinal prosthesis that serves to provide useful vision to people who are affected by retinal degenerative diseases such as retinitis pigmentosa (RP) [1, 2].

Retinitis pigmentosa (RP) is an inherited disease that is characterized by progressive degeneration of the retina, typically starting in the mid-periphery, and advancing toward the macula and fovea. The retina consists of both the outer retina, which contains photoreceptors (rods and cones), and the inner retina, which contains neural and glial cells. Initially in RP, there is progressive degeneration of the rod photoreceptor cells, which is then followed by degeneration of cone photoreceptor cells [3]. It is argued that the inner retinal cells survive degeneration, and therefore, serve as a site for electrical stimulation by the Argus II Retinal Prosthesis [4]. However, there is also evidence of retinal remodeling that occurs in the inner retinal neurons following degeneration of the outer photoreceptor layers. Structural changes that occur in the inner retinal layer include dendritic reorganization, as well as cell migration and layer disruption. Additionally, there are functional changes that occur, which includes changes in synaptic transmission and electrical coupling [3, 5]. Remodeling at the cellular level, as well as reprogramming that occurs at the molecular level, results in progressive neural degeneration limiting the ability for the inner layers to serve as areas of electrical stimulation.

The Argus II retinal prosthesis consists of a 6 × 10 array of platinum electrodes (diameter = 200 um) that is fixed to the retina-choroid-sclera with the use of a single retinal tack [6, 7]. According to Second Sight’s Surgical Manual, it is recommended that the electrode rows on the array be placed approximately at 45 degrees to the horizontal meridian. The ideal location being that the center of the electrode array is lined with the fovea [7]. The array is connected to an electronics case and implant coil that is wrapped around the eyeball using a scleral band. Additionally, there is an external portion of the device that is wirelessly connected to the implanted portion. The external part consists of a pair of glasses with a video camera allowing for real-time image capture, a video processing unit, and a coil on the sidearm of the glasses that allows for transmission of data using radiofrequency telemetry [2].

Limited research suggests that there are anatomical and electrode array positional changes post implantation. The position of the array over the macula region is noted to be necessary for optimal visual function [8]. Studies have shown that the distance between the electrode array and the retina changes over time due to macular thickening under the array [9, 10]. In addition, Delyfer et al. demonstrated in a study with 18 eyes that there is significant rotation of the electrode array in relation to the retina over a 6-month period postoperatively [9]. There are many factors that affect the position of the electrode array, such as surgical technique, the anatomy of the patient, and other pathological processes that may cause difficulty in reaching complete electrode apposition [9, 11–13].

This study aims to further analyze the implants’ positional stability over time. It primarily focuses on comparing the postoperative position of the retinal implant from month 1 to month 24 and to further analyze whether a rotation around the axis of the single tack occurs over time. We hypothesize that the electrode array changes in position over time and report such findings.

## Methods

### Study design

This study is a retrospective cohort, single center study. The study protocol was approved by the Institutional Review Board at Stony Brook University and is conducted in accordance with the tenets of the Declaration of Helsinki. Informed consent was obtained by the subjects. Patients enrolled in the Argus II Post-Approval Study (PAS) (clinicaltrials.gov identifier, NCT01860092) at Stony Brook University Hospital were invited to enroll in the study.

### Patient eligibility criteria

Inclusion criteria included: patients with profound retinal degeneration and subsequent vision loss resulting in bare light perception or worse vision (> 2.9 logMAR). Eligibility was extended to those with a diagnosis of outer retinal dystrophy. Patients are to attend follow-up visits, rehabilitation measures, and continuing device fitting and programming.

Exclusion criteria included: patients with diseases that could affect successful implantation and could compromise functional optic nerves, such as optic nerve disease, trauma, severe strabismus, central retinal artery or vein occlusion, history of retinal detachment. Patients with ocular conditions that could complicate surgical implantation were not included in the study, such as axial lengths < 20.5 mm or > 26 mm and/or corneal ulcers.

### Patient and public involvement

Patients or the public were not involved in the design, or conduct, or reporting, or dissemination plans of our research.

### Data collection

Patients were followed up at month 1 (M1), month 3 (M3), month 6 (M6), month 12 (M12), and month 24 (M24) post implantation. Fundoscopy was done at baseline prior to surgical implantation and at each subsequent follow up visit as part of the Argus II Retinal Prosthesis Post Approval Study. Images were taken using both Topcon 50 IX Retinal Camera (Topcon, Tokyo, Japan) and Optos Retinal Camera (Dunfermline, Scotland, United Kingdom).

### Postoperative Fundoscopy and image analysis

Fundoscopy from each follow-up visit (M1, M3, M6, M12 and M24) was extracted from the IBM Merge Healthcare (IBM Corp, Armonk, New York, USA) and Optos software using unique patient identifiers. As part of our study, each patient enrolled was identified as A01, A02, A03, A04 and A05 to maintain confidentiality. Images that were selected for study purposes at each visit consisted of clear depictions of the optic disc and the retinal implant. This allowed for the ability to analyze the data using NIH ImageJ software [14].

### Determination of linear and angular movement

**Fig. 1:**
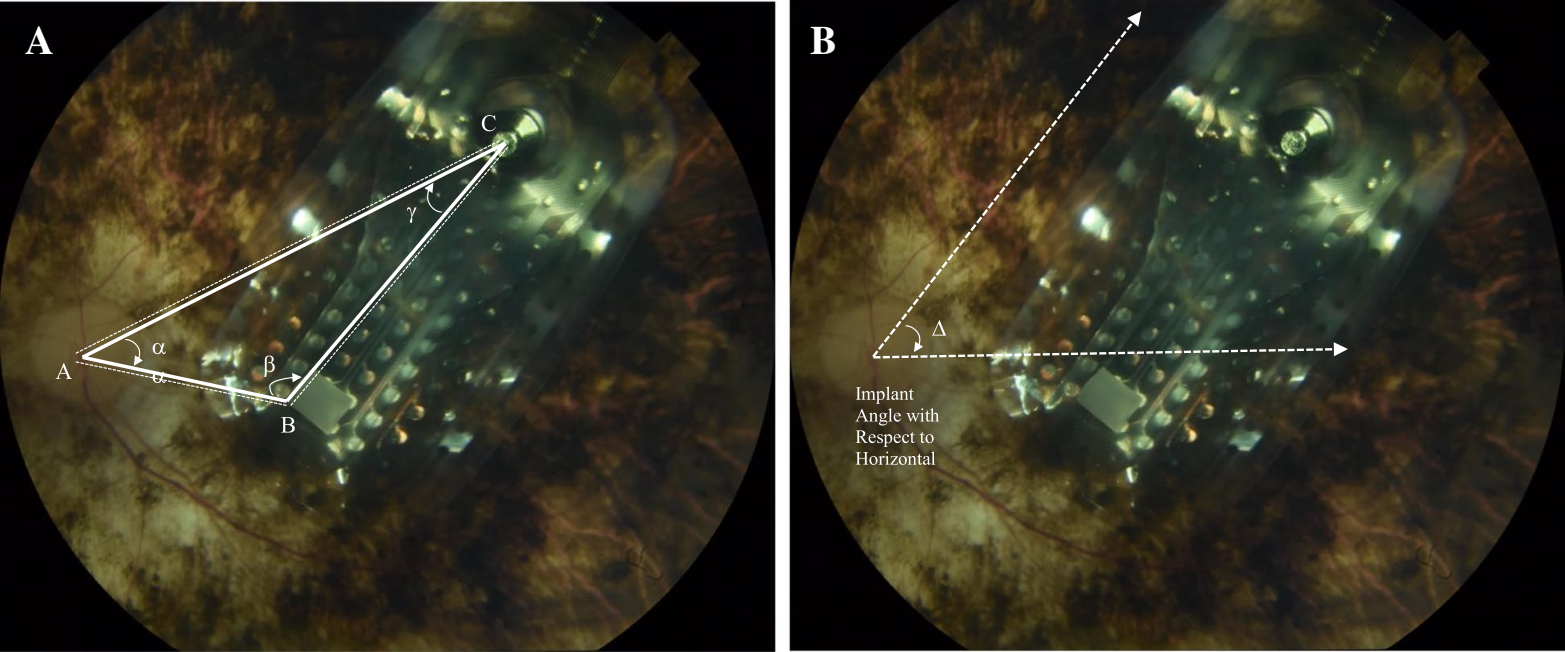
Fundoscopy of patient A-03 at month 24 post implantation with markings to show distances and angles that were recorded using NIH ImageJ: **A** A represents the point in the center of the optic disc, B is the point at the top corner of the surgical handle on the implant, C is the point in the center of the tack. Alpha (α) represents the ∠CAB, gamma (Ɣ) represents the ∠ACB, beta (β) represents ∠ABC. **B** delta (𝜟**)** measures the angle made by the line along the edge of the implant to the horizontal of the image frame

Fundus images from all patients taken during M1, M3, M6, M12, and M24 were imported into NIH ImageJ v1.53a to measure lengths AB, AC, BC, and angles **𝜶,** 𝜷, **𝛾,** and **𝜟** (Fig. 1) (Schneider et al. 2012). As reported by the Argus II Retinal Prosthesis System Surgeon Manual, the diameter of each electrode is 200 μm in diameter [7]. The diameter of one electrode from each image analyzed was measured in pixels on ImageJ, and a scale was set based on the 200-μm measurement provided by the surgeon manual. Following the calibration, each length was measured in micrometers, and angle measured in degrees.

Length AB represents the distance from the optic disc to the surgical handle on the electrode array. This distance reflects the implant moving towards or away from the optic disc in a linear fashion. Angle **𝛾** (optic disc-tack-surgical handle), angle **𝜶** (tack-optic disc-surgical handle), and angle 𝜷 (optic disc-surgical handle-tack) are reflective of rotation around the axis of the tack. Another measure for showing the rotation around the axis is the angle to the horizontal of the image frame (angle **𝜟**). Variations in such markers between postoperative visits M1, M3, M6, M12, and M24 would be reflective of the linear movement and rotation of the implant over time (Fig. 1).

Other markers recorded in this study were length AC which is the distance from the optic disc to the tack and length BC which is the distance from the surgical handle to the tack. Both are representative of steady measures over time and are used as retinal references.

### Statistical analysis

Repeated ANOVA was used to assess for changes in distance of the implant over time. *P* values less than or equal to 0.05 were considered statistically significant. All statistical analysis was conducted on IBM SPSS Version 27.0 (IBM Corp, Armonk, New York, USA).

## Results

### Participant demographics

Five participants were enrolled in this single center study under the supervision of a single vitreoretinal surgeon. All recruitment, surgeries and follow up visits were conducted at Stony Brook University Hospital and its affiliated sites (Stony Brook, New York). Demographics, diagnosis, age at implantation, implanted eye, axial length and the visual acuity at presentation are shown in Table 1.

**Table 1:** Participant demographics at baseline (*N* = 5)

No. of participants	5
Age at time of implantation (yrs)	69 [53-75]
Sex (M)	3 (60%)
Sex (F)	2 (40%)
Retinal degeneration diagnosis	
Leber congenital amaurosis	1 (20%)
Retinitis pigmentosa	4 (80%)
Bare light perception	5 (100%)
Implanted Eye (left eye (OS))	3 (60%)
Implanted Eye (right eye (OD)	2 (40%)
Axial length of implanted eye (mm)	23.0 [21.37-23.73]

Median [range] or N (%)

### Movement of the Implant’s electrode Array on the retina over time

**Fig. 2:**
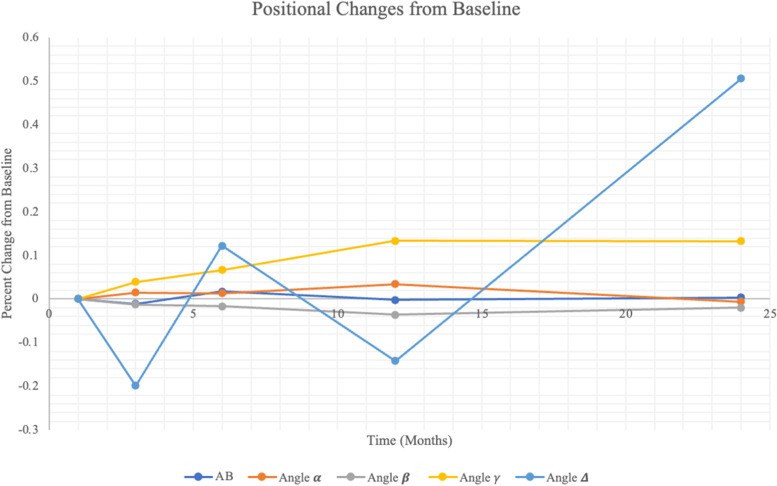
Positional changes of implant from baseline shown over 24 months

**Fig. 3:**
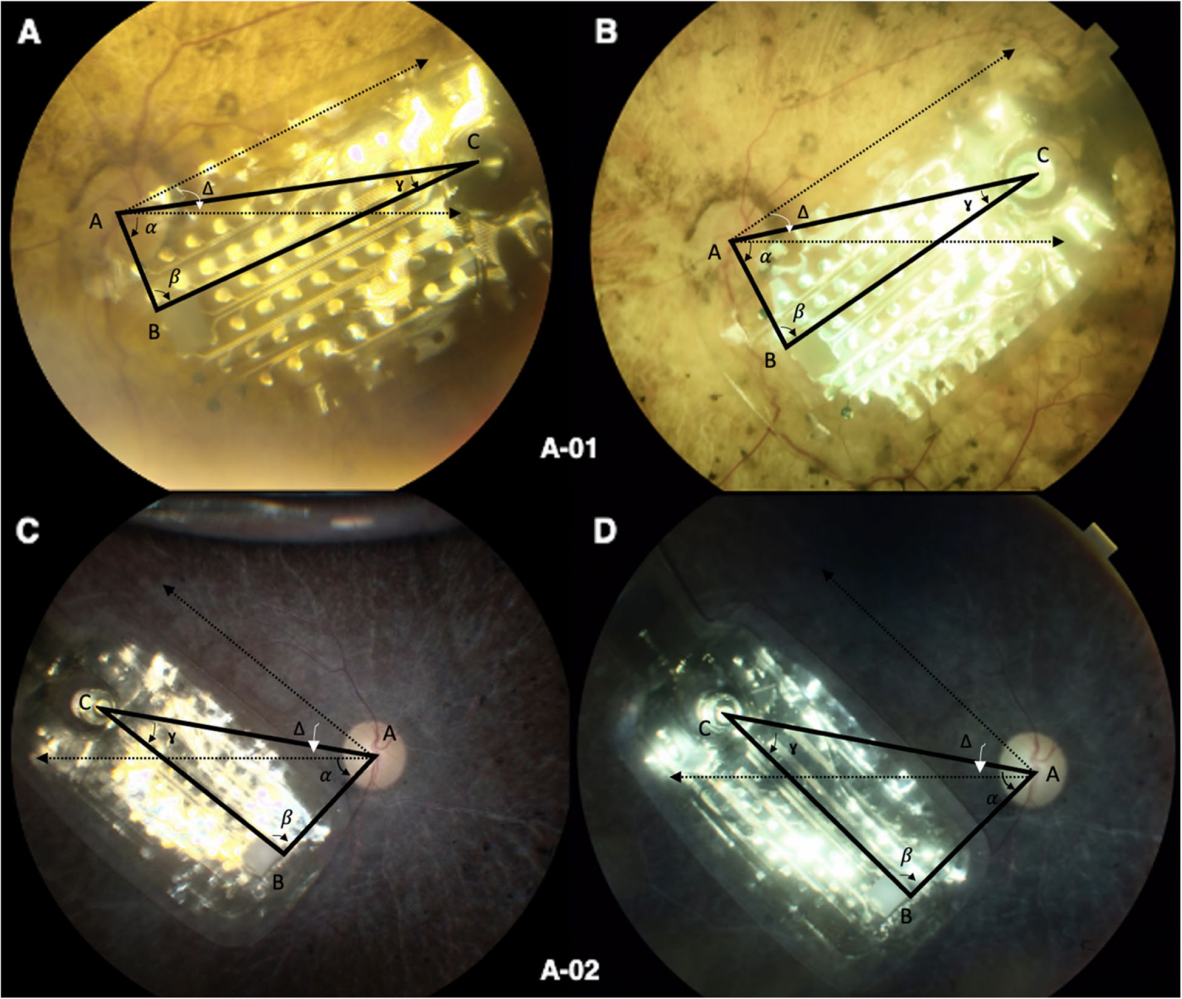
Color fundoscopy of (**A)** patient A-01 at M1, (**B**) patient A-01 at M24, (**C**) patient A-02 at M1, (**D**) patient A-02 at M24 with corresponding points and angles

The position of electrode array at M1, M3, M6, Y1 & Y2 is shown in Table 2. A one-way repeated measures ANOVA was performed to determine if there was statistically significant change in the mean movement of the implant changed over time (Figs. 2 and 3). Mauchly’s Test of Sphericity indicated that the assumption of sphericity had been violated for measures of length AB, X^2^(9) = 26.872, *p* = 0.005 and therefore, degrees of freedom were corrected using Greenhouse-Geisser estimate of sphericity ɛ = 0.279. A one-way repeated measures ANOVA revealed that there was no statistically significant difference in distance of length AB over time (M1 to M24) (*F* = 0.196, *p* = 0.705). Bonferroni’s test for multiple comparisons found that there was a significant difference in the change in distance over time between patients between M3 and M6 (*p* = 0.025).

**Table 2:** Position of electrode array at M1, M3, M6, Y1, & Y2

Length AB (μm)					
**Patient**	**M1**	**M3**	**M6**	**Y1**	**Y2**
**1**	**4699.0**	**4749.8 (+ 1.081%)**	**4978.4 (+ 5.946%)**	**4927.6 (+ 4.865%)**	**6070.6 (+ 29.189%)**
**2**	**7721.6**	**7594.6 (− 1.645%)**	**7747.0 (+ 0.329%)**	**7569.2 (− 1.974%)**	**7518.4 (− 2.632%)**
**3**	**6731.0**	**6553.2 (−2.642%)**	**6807.2 (+ 1.132%)**	**6654.8 (−1.132%)**	**5816.6 (− 13.585%)**
**4**	**7721.6**	**7442.2 (−3.618%)**	**7543.8 (− 2.303%)**	**7505.7 (−2.796%)**	**7632.7 (−1.151%)**
**5**	**7213.6**	**7366 (+ 2.113%)**	**7594.6 (+ 5.282%)**	**7353.3 (+ 1.937%)**	**7162.8 (−0.704%)**
**Average**	**6817.36**	**6741.16 (−1.118%)**	**6934.2 (+ 1.714%)**	**6802.12 (− 0.224%)**	**6840.22 (+ 0.335%)**
**(% change from position at M1)**					
**Angle 𝜶 (degrees)**					
**Patient**	**M1**	**M3**	**M6**	**Y1**	**Y2**
**1**	**78.487**	**78.323 (−0.209%)**	**76.211 (−2.900%)**	**75.655 (−3.608%)**	**70.81** **(−9.781%)**
**2**	**54.471**	**55.712 (+ 2.278%)**	**55.877 (+ 2.581%)**	**54.69(+ 0.402%)**	**54.876 (+ 0.744%)**
**3**	**38.707**	**39.189 (+ 1.245%)**	**39.308 (+ 1.553%)**	**39.643 (+ 2.418%)**	**38.534 (− 0.447%)**
**4**	**15.796**	**16.538 (+ 4.697%)**	**17.567 (+ 11.212%)**	**23.587 (+ 49.23%)**	**21.25 (+ 34.528%)**
**5**	**36.439**	**37.41 (2.665%)**	**37.85 (+ 3.872%)**	**37.961 (+ 4.177%)**	**36.978 (+ 1.479%)**
**Average**	**44.78**	**45.4344 (+ 1.461%)**	**45.3626 (+ 1.301%)**	**46.3072 (+ 3.410%)**	**44.4896 (−0.649%)**
**(% change from position at M1)**					
**Angle** 𝜷 **(degrees)**					
**Patient**	**M1**	**M3**	**M6**	**Y1**	**Y2**
**1**	**84.387**	**83.948 (−0.520%)**	**83.082 (−1.546%)**	**83.271 (− 1.322%)**	**87.474 (+ 3.658%)**
**2**	**97.598**	**94.05 (−3.635%)**	**91.652 (−6.092%)**	**91.9 (−5.838%)**	**92.42 (− 5.305%)**
**3**	**117.924**	**118.456 (0.451%)**	**119.707 (1.512%)**	**116.334 (−1.348%)**	**117.997 (+ 0.062%)**
**4**	**155.874**	**152.895 (−1.911%)**	**155.252 (−0.399%)**	**146.523 (−5.999%)**	**149.271 (−4.236%)**
**5**	**122.231**	**121.585 (−0.529%)**	**118.866 (−2.753%**	**119.244 (− 2.444%)**	**119.31 (− 2.390%)**
**Average**	**115.6028**	**114.1868 (−1.225%)**	**113.7118 (−1.636%)**	**111.4544 (−3.588%)**	**113.2944 (− 1.997%)**
**Angle 𝛾 (degrees)**					
**Patient**	**M1**	**M3**	**M6**	**Y1**	**Y2**
**1**	**17.126**	**17.729 (+ 3.521%)**	**20.707 (+ 20.910%)**	**21.074 (+ 23.053%)**	**21.716 (+ 26.801%)**
**2**	**27.931**	**30.238 (+ 8.260%)**	**32.471 (+ 16.254%)**	**33.41 (+ 19.616%)**	**32.704 (+ 17.089%)**
**3**	**23.369**	**22.355 (−4.339%)**	**20.985 (−10.202%)**	**24.023 (+ 2.799%)**	**23.469 (+ 0.428%)**
**4**	**8.33**	**10.567 (+ 26.855%)**	**7.181 (−13.794%)**	**9.89 (+ 18.727%)**	**9.479 (+ 13.794%)**
**5**	**21.33**	**21.005 (−1.524%)**	**23.284 (+ 9.161%)**	**22.795 (+ 6.868%)**	**23.712 (+ 11.167%)**
**Average**	**19.6172**	**20.3788 (+ 3.882%)**	**20.9256 (+ 6.670%)**	**22.2384 (+ 13.362%)**	**22.216 (+ 13.248%)**
**Angle 𝜟 (degrees)**					
**Patient**	**M1**	**M3**	**M6**	**Y1**	**Y2**
**1**	**28.936**	**35.352 (+ 22.173%)**	**34.789 (+ 20.227%)**	**34.226 (+ 18.282%)**	**39.783 (+ 37.486%)**
**2**	**37.126**	**41.952 (+ 12.999%)**	**44.068 (+ 18.698%)**	**43.516 (+ 17.212%)**	**44.599 (+ 20.129%)**
**3**	**47.003**	**45.433 (−3.340%)**	**52.836 (+ 12.410%)**	**46.701 (−0.643%)**	**54.716 (+ 16.410%)**
**4**	**32.373**	**30.683 (−5.220%)**	**33.573 (+ 3.707%)**	**31.017 (−4.189%)**	**37.504 (+ 15.850%)**
**5**	**38.873**	**43.603 (+ 12.168%)**	**40.862 (+ 5.117%)**	**40.687 (+ 4.666%)**	**38.748 (−0.322%)**
**Average**	**36.8622**	**39.4046 (+ 6.897%)**	**41.2256 (+ 11.837%)**	**39.2294 (+ 6.422%)**	**43.07 (+ 16.841%)**

Mauchly’s Test of Sphericity indicated that the assumption of sphericity was not violated for measures of angle **𝛾** over time, X^2^(9) = 10.652, *p* = 4.08 and therefore, we assumed sphericity. A one-way repeated measures ANOVA revealed that there was a statistically significant change of angle **𝛾** over time (M1 to M24) (*F* = 3.527, *p* = 0.030). Bonferroni’s test for multiple comparisons was done, but it did not show a significant difference in the change in distance between patients.

Mauchly’s Test of Sphericity indicated that the assumption of sphericity was not violated for measures of angle **𝜟** over time, X^2^(9) = 10.966, *p* = 0.385 and therefore, we assumed sphericity. A one-way repeated measures ANOVA revealed that there was no statistically significant change of angle **𝜟** over time (M1 to M24) (*F* = 1.797, *p* = 0.179). Bonferroni’s test for multiple comparisons was done but did not show a significant difference in the change in distance between patients.

Mauchly’s Test of Sphericity indicated that the assumption of sphericity had been violated for measures of angle **𝜶** over time, X^2^(9) = 26.844, *p* = 0.005 and therefore, degrees of freedom were corrected using Greenhouse-Geisser estimate of sphericity ɛ = 0.308. A one-way repeated measures ANOVA revealed that there was no statistically significant difference in distance of angle **𝜶** over time (M1 to M24) (*F* = 0.507, *p* = 0.546). Bonferroni’s test for multiple comparisons was done but did not show a significant difference in the change in distance between patients.

Mauchly’s Test of Sphericity indicated that the assumption of sphericity was not violated for measures of angle 𝜷 over time, X^2^(9) = 10.330, *p* = 0.433 and therefore, we assumed sphericity. A one-way repeated measures ANOVA revealed that there was no statistically significant change of angle 𝜷 over time (M1 to M24) (*F* = 2.556, *p* = 0.079). Bonferroni’s test for multiple comparisons was done but did not show a significant difference in the change in distance between patients.

In conclusion, repeated measures ANOVA showed that there was statistically significant change of the optic disc-tack-surgical handle angle (**𝛾**) (M1 to M24) (F = 3.527, p = 0.030). It showed that there was a significant change in the distance of length AB over time between patients from M3 to M6 (*p* = 0.025). However, there was no significant change in distance between the optic disc and the surgical handle (length AB) over the two-year span (*F* = 0.196, *p* = 0.705). There was also no significant change in angle 𝜟 (the angle to the horizontal of the image), angle 𝜶 (tack-optic disc-surgical handle), and angle 𝜷 (optic-disc-surgical handle-tack). The estimated marginal means of the angles and lengths of each measurement are shown in Fig. 2.

## Discussion

The Argus II Retinal Prosthesis aims to restore useful vision to patients affected by profound retinal degeneration [15]. Given the novelty of this device, and of retinal implants in general, there are many areas of the field that may be scientifically and medically observed and analyzed over time. There is data that suggests changes in retinal anatomy due to the implantation of such a device, and changes in the stability of the implants position over time [9]. Given that the electrode array is fixed to the retina by a single titanium tack, it may be possible to observe movement over time. Reported in a study by Delvfer et al., this hypothesis was confirmed by a slight and significant rotation of the array between months 1 and 6 postoperative follow up visits [11].

Our study aims to further such analysis by observing both linear and angular movement of the electrode area over a two-year postoperative period. It was found that the prosthetic displays both linear and rotational movement, post-operatively, in a non-random fashion.

Analysis of fundus images at each patient’s routine post-operative appointments revealed that the electrode array was shifting in its position in both a linear and rotational fashion between each appointment. Notably, there was a significant increase in the mean linear distance from the optic disc to the surgical handle on the implant (distance AB) with patients between month 3 and month 6, with a mean shift of 193.04 μm in a general downward direction. There was additionally a significant increase in the angle between the optic disc and the implant surgical tack (**𝛾)**, notable throughout the two-year period. This movement indicates a counterclockwise rotation around the implant’s surgical tack over time postoperatively.

A possible explanation to the downward rotation of the electrode array can be the effect of gravity over time. Gravity could produce a downward force causing the implant to shift in position. Additionally, eye rubbing is contraindicated in patients who are eligible in receiving the retinal prosthesis because this could result in device exposure and erosion [2]. Therefore, if the patients were to rub their eyes for any reason, this mechanical movement of the eye could result in the shift of the implant. There may also be an inherent “memory” within the array cable leading to the intraocular linear and rotational change over time. Furthermore, saccadic eye movements may contribute to the shift of the implant.

Cruz et al. demonstrated, in a study analyzing the reliability and stability of the retinal implant over 5 years, that there were two devices (out of thirty) that failed [1]. The devices were implanted safely, according to the respective guidelines, but were deemed nonfunctional due to dysfunctional radiofrequency links between the internal antenna on the array and the external antenna on the glasses. The study describes that the devices failed most likely due to exposure of a portion of the receiving antenna [1]. Such exposure could be due to the instability, and consequently, the movement of the implant over time. Furthermore, titanium retinal tacks were previously used for surgical treatment of retinal detachments. They were found to cause irritation of the retina resulting in glial reactions [2, 11, 16, 17]. Such traction from fibrosis can also potentially result in the shift of the implant over time.

One patient (patient 1) in our study developed clinically significant retinoschisis. This patient had almost complete apposition of the implant on the retina and it has been hypothesized that complete apposition may increase the risk of adverse postoperative outcomes due to traction of the implant on the retina, increased inflammation resulting in remodeling of the retinal layers and/or overstimulation of the retina [10]. Such factors may also increase the risk of the implant’s movement.

Given that the retinal implant is fixated by a single tack, theoretically, movement of the implant may occur over time. In our study, we note that all patients saw a change in length AB over time, with patient 1 showing a major change of 1320.9 μm compared to the M1 (baseline) measurement. The electrodes are each 200 μm and the entire array covers about 20 degrees of visual angle diagonally. The array covers an area on the retina that corresponds to 18° × 11°, assuming 293 μm corresponds to 1 degree of visual angle [18]. Given this standard, the change in length AB for patient 1 corresponds to 4.51 degrees of visual angle. It has been shown in previous studies that visual precepts can range depending on the retinal location of the stimulating electrode. Such epiretinal stimulation can activate passing axon fibers which can distort the quality of a patient’s visual outcomes. A computational model done by a study published by Beyeler et al. showed that such distortions are related to the topographic organization of optic nerve fiber bundles in the patient’s retina [19]. For patient 1, given a 4.51 degree of visual angle movement, the phosphene shape may change resulting in significant visual outcome distortion.

Additionally, a single electrode within the implant leads to activation of a wide variety of distinct retinal cells, encompassing hundreds of photoreceptors. In severe end-stage retinitis pigmentosa, little to no useful vision is retained. With progression of the disease, relative amounts of surviving bipolar and ganglion cell types may vary, influencing phosphene shape [19]. Therefore, movement of the implant may change phosphene shapes as perceived by patients, and thus, affecting visual outcomes for patients. However, the significance of the effect may be difficult to ascertain without formal testing. Future studies with a larger data set may be able to explore the changes in visual outcome changes because of the movement of the Argus II Retinal Prosthesis implant over time.

Given that this study recruited patients from a single institution through a single surgeon, it was underpowered by its inability to recruit more than five patients. We believe that comparing the changes over time with a small sample size may have resulted in type I error and therefore we may not be able to create definite conclusions regarding the data. However, to the authors knowledge, it is the first time angular and linear movement of the array is described in a series of patients over a two-year period. Future studies may recruit a larger sample to further analyze the results of this study and explore the relationship between postoperative movement of the implant with functional vision outcomes.

## Conclusion

The results of this study demonstrate that there is both statistically significant linear implant movement and rotation around the axis of the implant tack over time in patients with the Argus II Retinal Prosthesis System. This study demonstrates the importance of considering anatomic changes that occur upon implantation of a retinal prosthesis, and that an implant’s efficacy can be affected by anatomic changes. It is important, moving forward, to take this into consideration when designing retinal implants. Future studies may investigate the effects of this movement on patient outcomes over time due to positional changes.

## Data Availability

The datasets used and/or analyzed during the current study are available from the corresponding author on request.
